# A Mutation in the *FAM83G* Gene in Dogs with Hereditary Footpad Hyperkeratosis (HFH)

**DOI:** 10.1371/journal.pgen.1004370

**Published:** 2014-05-15

**Authors:** Michaela Drögemüller, Vidhya Jagannathan, Doreen Becker, Cord Drögemüller, Claude Schelling, Jocelyn Plassais, Cécile Kaerle, Caroline Dufaure de Citres, Anne Thomas, Eliane J. Müller, Monika M. Welle, Petra Roosje, Tosso Leeb

**Affiliations:** 1 Institute of Genetics, Vetsuisse Faculty, University of Bern, Bern, Switzerland; 2 DermFocus, University of Bern, Bern, Switzerland; 3 Clinic for Reproductive Medicine, University of Zurich, Zurich, Switzerland; 4 CNRS, UMR 6290, Institut Génétique et Développement de Rennes, Rennes, France; 5 Université Rennes 1, UEB, Biosit, Faculté de Médecine, Rennes, France; 6 Antagene, Animal Genetics Laboratory, La Tour de Salvagny, France; 7 Institute of Animal Pathology, Vetsuisse Faculty, University of Bern, Bern, Switzerland; 8 Division of Clinical Dermatology, Vetsuisse Faculty, University of Bern, Bern, Switzerland; Stanford University School of Medicine, United States of America

## Abstract

Hereditary footpad hyperkeratosis (HFH) represents a palmoplantar hyperkeratosis, which is inherited as a monogenic autosomal recessive trait in several dog breeds, such as e.g. Kromfohrländer and Irish Terriers. We performed genome-wide association studies (GWAS) in both breeds. In Kromfohrländer we obtained a single strong association signal on chromosome 5 (p_raw_ = 1.0×10^−13^) using 13 HFH cases and 29 controls. The association signal replicated in an independent cohort of Irish Terriers with 10 cases and 21 controls (p_raw_ = 6.9×10^−10^). The analysis of shared haplotypes among the combined Kromfohrländer and Irish Terrier cases defined a critical interval of 611 kb with 13 predicted genes. We re-sequenced the genome of one affected Kromfohrländer at 23.5× coverage. The comparison of the sequence data with 46 genomes of non-affected dogs from other breeds revealed a single private non-synonymous variant in the critical interval with respect to the reference genome assembly. The variant is a missense variant (c.155G>C) in the *FAM83G* gene encoding a protein with largely unknown function. It is predicted to change an evolutionary conserved arginine into a proline residue (p.R52P). We genotyped this variant in a larger cohort of dogs and found perfect association with the HFH phenotype. We further studied the clinical and histopathological alterations in the epidermis *in vivo*. Affected dogs show a moderate to severe orthokeratotic hyperplasia of the palmoplantar epidermis. Thus, our data provide the first evidence that FAM83G has an essential role for maintaining the integrity of the palmoplantar epidermis.

## Author Summary

The palms and soles of mammals are covered by the palmoplantar epidermis, which has to bear immense mechanical forces and has therefore a special composition in comparison to the epidermis on regular skin. We studied a Mendelian disease in dogs, termed hereditary footpad hyperkeratosis (HFH). HFH affected dogs develop deep fissures in the paw pads, which are the consequence of a pathological thickening of the outermost layer of the epidermis. We mapped the disease causing genetic variant in the Kromfohrländer and Irish Terrier breeds to a 611 kb interval on chromosome 5. HFH affected Kromfohrländer and Irish Terriers shared the same haplotype indicating descent from a common founder. We re-sequenced the genome of an affected dog and compared it to genome sequences of 46 control dogs. The HFH affected dog had only one private non-synonymous variant in the critical interval, a missense variant of the *FAM83G* gene. We genotyped this variant in more than 500 dogs and found perfect association with the HFH phenotype. Our data very strongly suggest that the *FAM83G* variant is causative for HFH. FAM83G is a protein with unknown biochemical function. Our study thus provides the first link between this protein and the palmoplantar epidermis.

## Introduction

The skin and most notably its outermost layer, the epidermis, forms an essential barrier against the environment. The soles of the feet and the palms of the hands are covered by the specially structured palmoplantar epidermis, which has to bear the strongest mechanical forces of the entire skin. Epidermolytic palmoplantar keratoderma (EPPK) is an inherited disorder characterized by abnormal thickening of the palmoplantar epidermis. It is typically caused by dominant variants in the *KRT9* gene encoding keratin 9, a type I intermediate filament specifically expressed in the suprabasal layer of the palmoplantar epidermis [1], [2]. Most human EPPK patients are heterozygous for dominant *KRT9* variants [1]. However, homozygous *Krt9* deficient mice show a very similar phenotype [2]. Related human genodermatoses which may involve the palmoplantar epidermis, but are not exclusively restricted to palms and soles are caused by variants in *KRT1* [3], *KRT10* [4], *KRT16* [5]–[7], and *AQP5* encoding the water channel aquaporin 5 [8]. Many genetic defects in the keratin genes themselves have been characterized in keratinizing disorders and provided first insights into the function of specific keratins in the various epithelia. However, much less is known about other molecules that interact with the keratins and are potentially involved in posttranslational modifications of keratins, or other regulatory mechanisms ensuring the mechanical stability of the epidermis [9], [10].

Spontaneous animal mutants with genodermatoses or other heritable phenotypes of the skin provide an opportunity to identify further components of the complex molecular machinery required to maintain skin function. Due to their special population structure purebred dogs are particularly well suited for genetic analyses [11]. Successful examples for the utilization of dog genetics in skin research include the identification of genes involved in the determination of hair characteristics [12], ectodermal development [13], one form of ichthyosis [14], congenital keratoconjunctivitis sicca and ichthyosiform dermatosis [15], the excessive skin folding in Chinese Shar Pei dogs [16], and hereditary nasal parakeratosis [17].

Hereditary footpad hyperkeratosis (HFH, also known as digital hyperkeratosis (DH) or “corny feet”) is a specific form of an orthokeratotic palmoplantar hyperkeratosis, which has been originally described in Irish Terriers [18]. HFH has also been observed in other related dog breeds, such as e.g. the Kromfohrländer, a young German dog breed founded in 1945. HFH initially leads to thickened and hardened footpads, which can be recognized in juvenile dogs starting at an age of 18 to 24 weeks. The inelastic pad surface subsequently develops cracks and fissures, which predispose affected dogs to secondary infections. If not properly managed, HFH can thus lead to considerable pain and lameness in affected dogs. HFH is inherited as a monogenic autosomal recessive trait [18]. A previous candidate gene study was not successful in identifying the causative gene [19].

In this study we used genome-wide association studies (GWAS) in independent Kromfohrländer and Irish Terrier cohorts and whole genome re-sequencing (WGS) to identify the causative genetic lesion for HFH in both breeds.

## Results

### Mapping of the causative mutation

**Figure 1:**
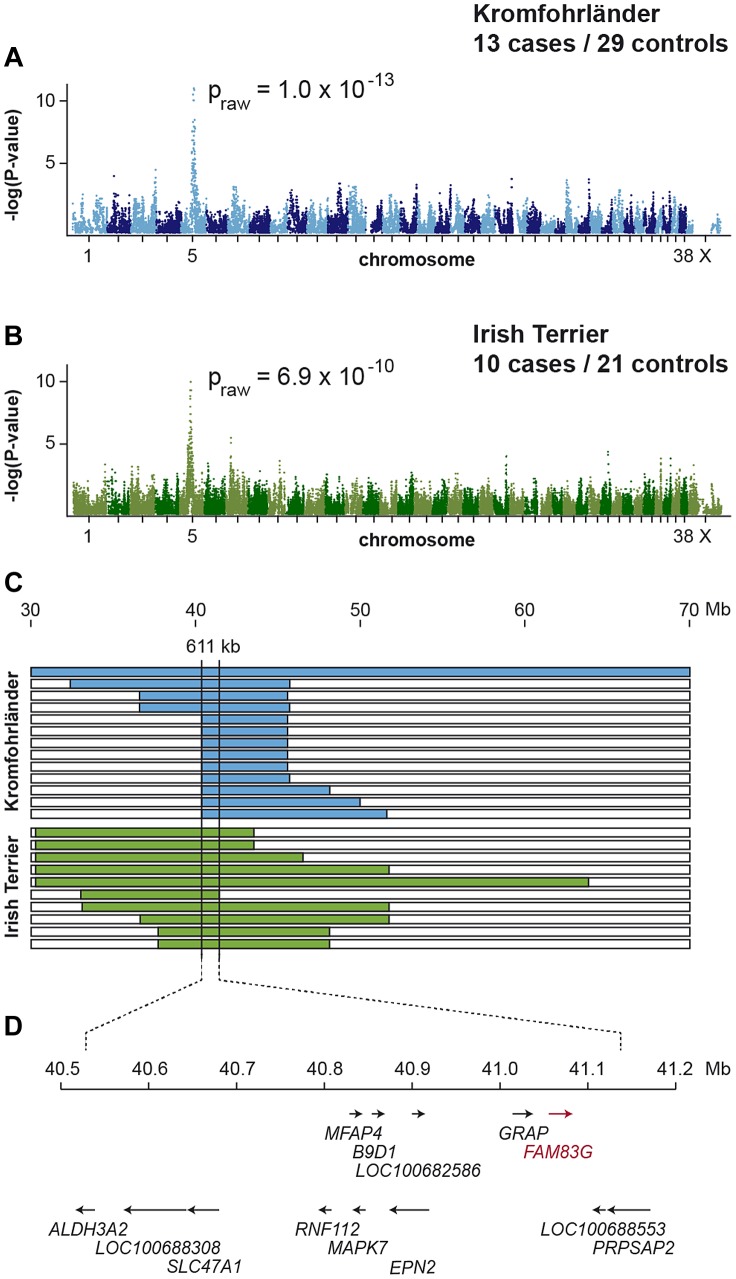
Mapping of HFH by GWAS and haplotype analysis. (A) A genome-wide association study in Kromfohrländer using 13 cases and 29 controls indicates a strong signal with multiple associated SNPs on CFA 5. (B) The association is replicated in a cohort of 10 Irish Terrier cases and 21 controls. (C) Homozygosity mapping. Each horizontal bar corresponds to one of the 23 analyzed cases. Homozygous regions with shared alleles are shown in color. A shared homozygous interval of 611 kb delineates the exact boundaries of the critical interval from 40,521,040–41,131,739 (CanFam 3.1 assembly). (D) NCBI gene annotation of the CanFam3.1 assembly in the critical interval.

We collected samples from 13 HFH affected Kromfohrländer and 29 controls and genotyped them with the 173k SNP chip. After removing 95,759 markers, which had low call rates (<90%), were non-informative (MAF <0.05), or showed a strong deviation from Hardy-Weinberg equilibrium in the controls (p<10^−5^), we retained 77,903 markers for the final genome-wide allelic association study. Three best-associated SNPs in the GWAS had identical raw p-values of 1.0×10^−13^ (Figure 1A). The corrected p-value after 100,000 permutations was <10^−5^. The 159 best-associated SNPs with raw p-values of less than 1×10^−4^ were all located on chromosome 5. The genomic inflation factor in this analysis was 1.40.

We also performed a GWAS in an independent cohort of Irish Terriers to replicate our findings. For the replication we had 10 cases, 21 controls, and 82,671 markers. In the Irish Terrier cohort HFH was also strongly associated with the same region on chromosome 5 with a raw p-value of 6.9×10^−10^ (Figure 1B). The genomic inflation factor in the Irish Terrier analysis was 1.32. As both cohorts showed considerable population stratification we repeated the analyses with a mixed model that corrects for this confounding effect. The same markers as in the initial analyses showed the strongest associations.

Subsequently, we applied a homozygosity mapping approach to fine-map the region containing the HFH mutation. We hypothesized that the affected dogs most likely were inbred to one single founder animal. In this scenario, the affected individuals were expected to be identical by descent (IBD) for the causative mutation and flanking chromosomal segments. We analyzed the 23 combined cases for extended regions of homozygosity with simultaneous allele sharing. In the associated interval on CFA 5, all 23 affected dogs were homozygous and shared identical alleles over 36 consecutive SNP markers. We concluded that the causative mutation should be located in the 611 kb critical interval between the closest heterozygous markers on either side of the homozygous segment (CFA5: 40,521,040–41,131,739 CanFam 3.1 assembly; Figure 1C).

### Mutation identification

A total of 13 genes and loci are annotated in the critical interval on CFA 5 (Figure 1D). In order to obtain a comprehensive overview of all variants in the critical interval we sequenced the whole genome of one affected Kromfohrländer. We collected nearly 293 million paired-end reads from a shotgun fragment library corresponding to 23.5× coverage of the genome. We called SNPs and indel variants with respect to the reference genome of a presumably non-affected Boxer. Across the entire genome, we detected ∼6.8 million variants of which ∼3.5 million were homozygous (Table 1). Within the critical interval there were 1,314 variants of which 16 were predicted to be non-synonymous.

**Table 1:** Variants detected by whole genome re-sequencing of an affected Kromfohrländer.

Filtering step	Number of variants
Variants in the whole genome^a^	3,528,020
Variants in the critical 611 kb interval on CFA 5	1,314
Variants in the critical interval that were absent from 46 other dog genomes	2
Non-synonymous variants in the whole genome^a^	11,437
Non-synonymous variants in the critical 611 kb interval on CFA 5	16
Non-synonymous variants in the critical interval that were absent from 46 other dog genomes	1

a The sequences were compared to the reference genome (CanFam 3.1) from a Boxer. Only variants that were homozygous in the affected Kromfohrländer are reported.

**Figure 2:**
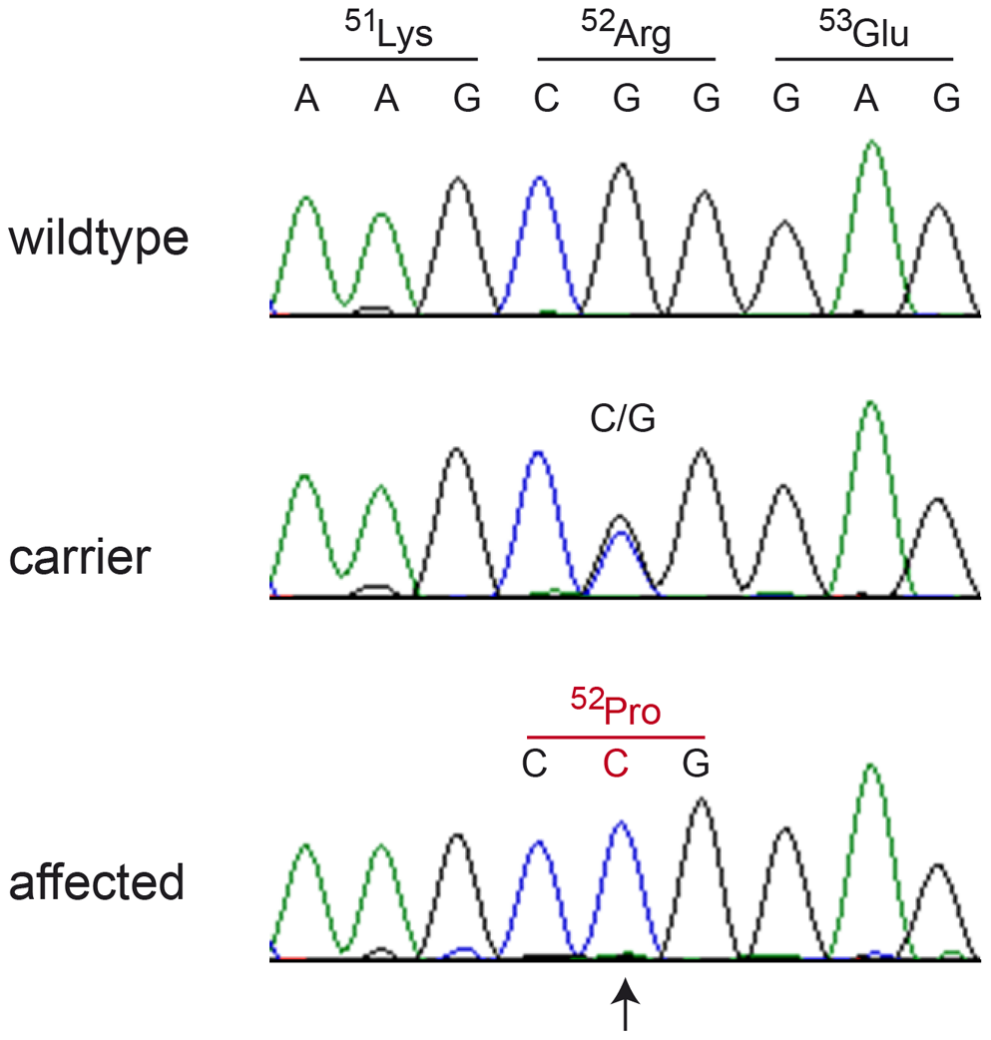
Electropherograms of the *FAM83G:c.155G>C* variant. A fragment harboring exon 2 and flanking sequences of the *FAM83G* gene was PCR-amplified and sequenced with the Sanger method. The figure shows representative traces from Kromfohrländer with the 3 different genotypes. The position of the variant is indicated by an arrow.

We further compared the genotypes of the affected Kromfohrländer with 46 dog genomes of various breeds that had been sequenced in the course of other ongoing studies (Table S1). We hypothesized that the mutant allele at the causative variant should be completely absent from all other dog breeds in our sample set. After this filtering step only two private variants remained in the critical interval and only one of them was predicted to be non-synonymous, *FAM83G:c.155G>C* or Chr5:41,055,619G>C. We confirmed this variant by Sanger sequencing (Figure 2) and genotyped it in 43 Kromfohrländer, 194 Irish Terriers, and 288 dogs of other breeds. It was perfectly associated with the HFH phenotype (Table 2, Table S2).

**Table 2:** Association of the *FAM83G:c.155G>C* variant with the HFH phenotype.

Genotype	Kromfohrländer cases	Kromfohrländer controls	Irish Terrier cases	Irish Terrier controls	Dogs from other breeds
*FAM83G:c.155G>C*					
* G/G*	-	21	-	148	288
* C/G*	-	9	-	23	-
* C/C*	13	-	23	-	-

**Figure 3:**
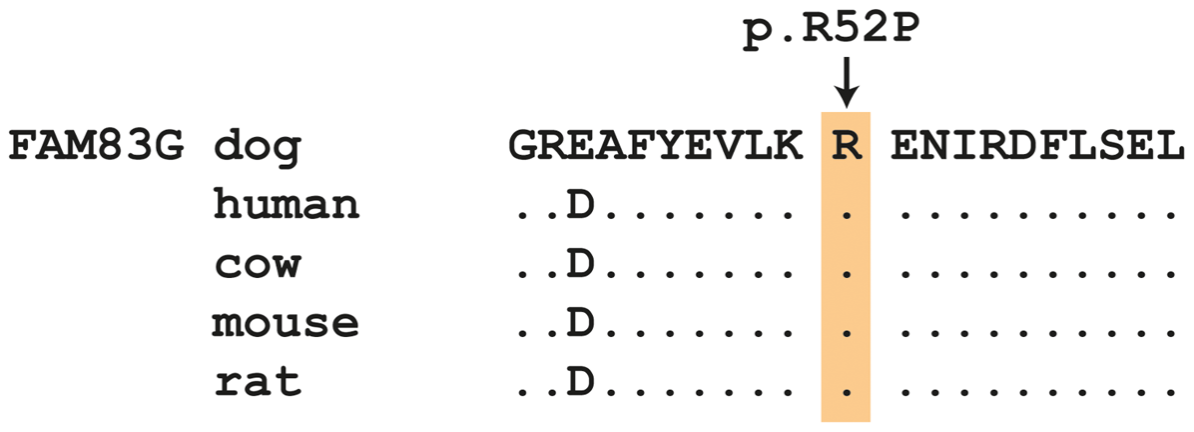
Evolutionary conservation of the arginine residue at position 52 in the FAM83G protein. All mammals share identical amino acid sequences in the region of the variant. The sequences were derived from the following database accessions: *C. lupus* XP_003434684.1, *H. sapiens* NP_001035088.2, *B. taurus* NP_001192445.1, *M. musculus* NP_848733.2, *R. norvegicus* XP_002727764.1.

The *FAM83G:c.155G>C* variant represents a missense mutation in the *FAM83G* gene, encoding the family with sequence similarity 83, member G. The variant changes an arginine codon to a proline codon (p.R52P). SIFT, Polyphen-2, and PMUT predict that this non-conservative amino acid exchange affects protein function [20]–[22]. The arginine at position 52 is perfectly conserved across all known eutherian FAM83G orthologs (Figure 3). We confirmed by immunofluorescence that FAM83G is strongly expressed in footpad epidermis, but not the underlying dermis (Figure S1).

### Refinement of the phenotype

**Figure 4:**
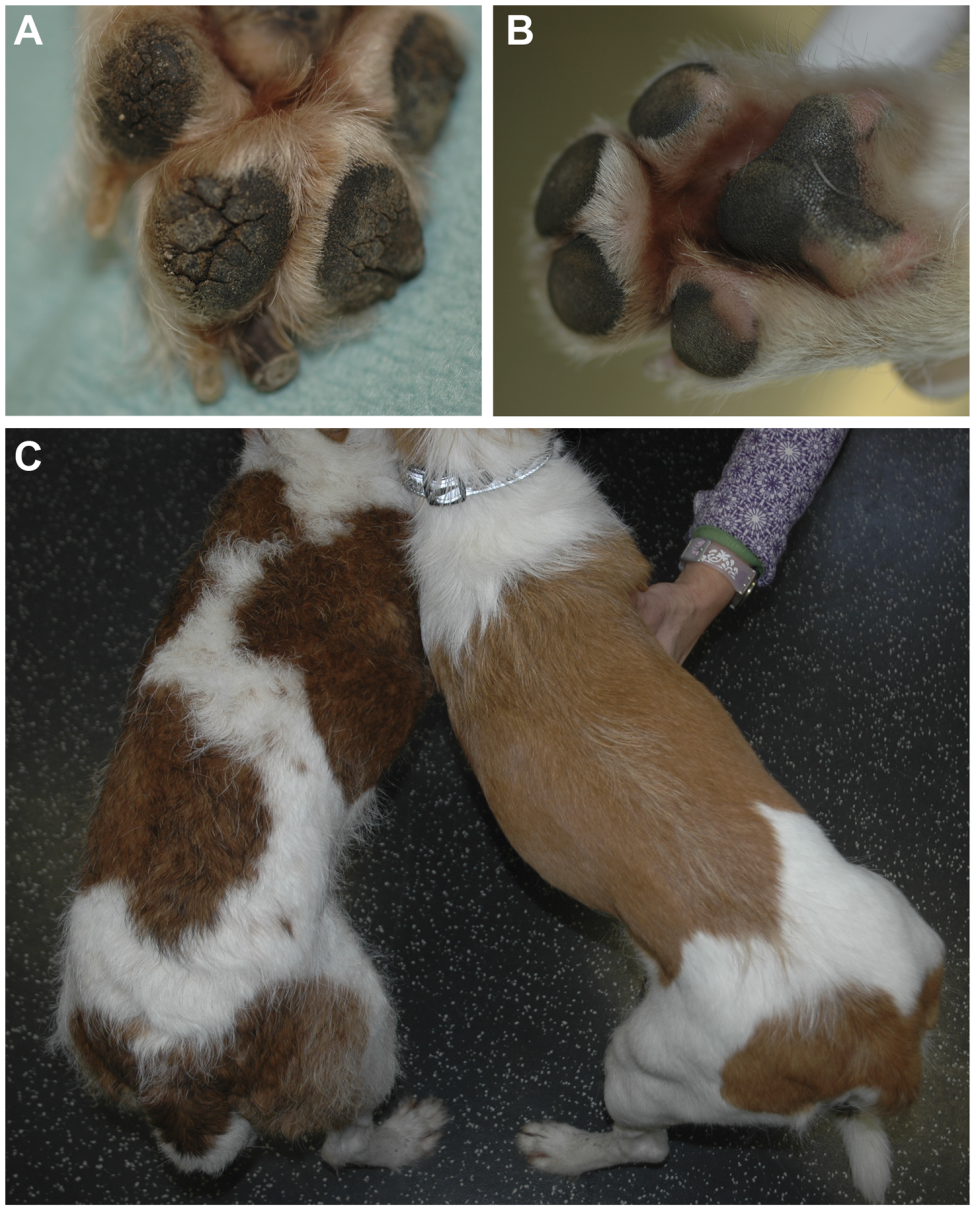
Clinical phenotype of digital hyperkeratosis. (A) Paw of a 1 year old affected Kromfohrländer. Note the cracked surface and deep fissures of the foot pads. (B) Paw of a control Kromfohrländer. (C) Hair coat of the dogs shown in panels A and B. The affected dog (left) has a more irregular coat appearance in comparison to the unaffected control dog (right). Both dogs are representatives of the wire-haired (“rough-coated”) Kromfohrländer variety.

Hyperkeratosis of the foot pads is noticed by the owners of both breeds at 4–5 months of age and involves all footpads. With time horny protrusions appear on the rims of the footpads and the pad surface becomes hard and develops cracks (Figure 4A). Affected animals avoid walking on irregular surfaces and may go lame. The nails of affected dogs are very hard and seem to grow faster. We noticed a duller, less wiry, softer coat on an affected Kromfohrländer (Figure 4C). Similar clinical symptoms were noted on 5 HFH affected Irish Terriers.

**Figure 5:**
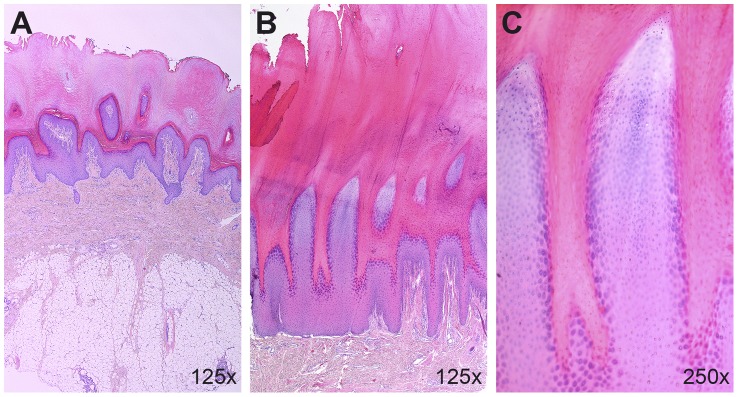
Histopathological findings in the palmoplantar epidermis. (A) Haematoxylin and eosin (HE) stained paw pad section from a non-affected control dog. Note the relatively thin layer of orthokeratotic keratin on the epidermal surface. (B) Paw pad of a 1 year old affected Kromfohrländer. Note the moderate epidermal hyperplasia with papillated epidermal protrusions to the outside. The epidermis is covered by abundant compact orthokeratotic keratin. (C) Higher magnification of the same biopsy as shown in panel B. Note that the epidermis is regularly differentiated and no nuclei are seen in the stratum corneum.

We also performed histopathological examinations of palmoplantar and normal epidermis. We did not observe any obvious changes in the normal epidermis from an HFH affected Kromfohrländer (data not shown). A paw pad biopsy from an affected Kromfohrländer revealed a moderate to severe palmoplantar epidermal hyperplasia associated with papillated epidermal protrusions to the outside. The differentiation of the dermal keratinocytes was morphologically normal. The palmoplantar epidermis was covered by abundant compact orthokeratotic keratin (Figure 5).

## Discussion

In this study we identified a missense variant of FAM83G as candidate causative genetic defect for HFH in two related dog breeds. We cannot formally rule out the possibility that another, potentially non-coding regulatory variant, in absolute linkage disequilibrium (LD) with the *FAM83G:c.155G>C* variant is the actual causative variant. However, in our genome re-sequencing data, there is only one other variant in complete LD with the *FAM83G* variant. This variant is an intergenic SNP more than 15 kb away from the next annotated transcript and thus unlikely to be functionally important.

It also has to be considered that our variant detection relied on short read mapping to an imperfect reference genome. Thus, we will have missed variants, which are located in genome segments that are not contained in the reference genome, such as gap regions. We may also have missed non-synonymous variants in genes that are not or not correctly annotated in the dog reference genome.

While acknowledging the limitations of the currently available technologies our genome re-sequencing data taken together with the precise genetic mapping and the strictly recessive mode of inheritance of HFH, which suggests a complete loss-of-function allele, very strongly support the causality of *FAM83G:c.155G>C*.

*FAM83G* is hardly characterized so far. It has recently been shown that a partial deletion of the *Fam83g* gene causes the phenotype of the wooly (*wly*) mouse mutant [23]. Wooly mice macroscopically display a rough or matted appearance of their coat. Similar to our findings in dogs and in spite of this clearly visible macroscopic phenotype, microscopic examination did not reveal any consistent anomaly in any of the four murine hair types nor any consistent changes in skin histology [23], [24]. According to our knowledge the foot pads of wooly mice have not been studied in detail.

The FAM83 protein family consists of 8 known members FAM83A – FAM83H. Apart from the single report on murine Fam83g, a physiological *in vivo* function has so far only been discussed for FAM83H [15], [23], [25]. Heterozygous nonsense variants in the last exon of the *FAM83H* gene have been reported in human patients with autosomal dominant hypocalcified ameliogenesis imperfecta, a disorder of enamel formation during tooth development [25]. However, a *FAM83H* frameshift variant in the last exon was shown to cause the autosomal recessive congenital keratoconjunctivitis sicca and ichthyosiform dermatosis (CKCSID), also called “dry eye curly coat syndrome”, in the Cavalier King Charles Spaniel dog breed [15]. The apparent discrepancy in the reported phenotypes of human and dog patients with *FAM83H* variants might be due to the specific nature of the involved variants and calls for further investigations. In this context, it is interesting to note that the *FAM83H* mutant Cavalier King Charles Spaniels have a defect that shares several phenotypic features with the *FAM83G* mutant Kromfohrländer and Irish Terriers, such as an altered coat texture, altered nails and palmoplantar hyperkeratosis.

The FAM83 members share a conserved protein domain of about 300 amino acids at their N-terminus (Pfam DUF1669), which shows homology to the phospholipase D catalytic domain. However, as critical catalytic histidine residues are lacking, it is unlikely that this domain actually has a phospholipase activity in the members of the FAM83 family [23].

FAM83G is highly conserved in eutherians (placental mammals). In more distantly related vertebrate species the predicted FAM83G orthologs show a drastically reduced overall homology of the amino acid sequence, which might indicate that FAM83G acquired a new function during eutherian or possibly mammalian evolution. It is tempting to speculate that this new function is related to the evolution of hair and a specialized palmoplantar epidermis in mammals.

In conclusion, we have identified a missense variant in *FAM83G* as most likely causative for HFH in dogs. This provides a first indication of a physiological function of this particular gene in maintaining the integrity of the palmoplantar epidermis. Together with previous data from mice our data also confirm that this gene has an additional role in hair morphology.

## Materials and Methods

### Ethics statement

All animal experiments were performed according to the local regulations. The dogs in this study were examined with the consent of their owners. The study was approved by the “Cantonal Committee For Animal Experiments” (Canton of Bern; permit 23/10).

### Animal selection and phenotype assignment

We used 13 HFH cases and 30 controls from the Kromfohrländer breed. The phenotype information was extracted from a database that is maintained by the breeding club and based on reports from dogs' owners and evaluations by the breeding committees of the club. One of the 13 Kromfohrländer cases was additionally examined by a board certified veterinary dermatologist (PR; Figure 4) and the clinical diagnosis was confirmed by the histhopathological analysis of a biopsy from the footpad, which was evaluated by a board certified veterinary pathologist (MMW; Figure 5).

In the Irish Terrier breed we initially started our analysis with 26 reported cases and 171 controls. In the Irish Terriers we also primarily relied on phenotypes as reported by the owners. We initially selected 13 owner-reported cases and 21 owner-reported controls for the GWAS and homozygosity mapping. During this analysis we realized that 3 of the owner-reported cases did not carry the disease-associated haplotype. This prompted us to carefully re-evaluate the phenotypes of all Irish Terrier cases. It then turned out that the 3 suspicious dogs had only lesions on one to three feet, whereas all other Irish Terrier cases had lesions on the footpads of all four feet. We therefore used a refined phenotype classification, which required reported lesions on all four feet for HFH cases. Based on this new phenotype classification we assumed the 3 Irish Terriers that did not have lesions on all four feet to represent phenocopies and excluded them from all further analyses.

We thus ended up with 23 Irish Terrier HFH cases and 171 controls for the final analysis. We also used additional DNA samples from other breeds that were collected for various research projects at the Institute of Genetics of the University of Bern. For these other samples a non-affected phenotype was assumed as HFH is supposedly confined to the Kromfohrländer and Irish Terrier breeds.

### DNA samples and SNP genotyping

We isolated genomic DNA from EDTA blood samples with the Nucleon Bacc2 kit (GE Healthcare) and from cheek swabs with the NucleoSpin 96 Tissue DNA Kit (Macherey-Nagel). Genotyping was done on illumina canine_HD chips by GeneSeek/Neogen (Kromfohrländer, 173,662 SNPs called) or the Centre National de Génotypage, Evry, France (Irish Terriers, 174,376 SNPs called). Genotypes were stored in a BC/Gene database version 3.5 (BC/Platforms).

### Genome-wide association study (GWAS) and homozygosity mapping

We used PLINK v1.07 [26] to perform genome-wide association analyses (GWAS). In the Kromfohrländer analysis all 13 cases and 29 controls had call rates >90%. We removed 3,303 markers with call rates <90% from the analysis. We also removed 94,714 markers with minor allele frequency (MAF) <5% and 30 markers strongly deviating from Hardy-Weinberg equilibrium in the controls (p<10^−5^). The final Kromfohrländer dataset consisted of 42 dogs and 77,903 SNPs.

In the Irish Terrier cohort with 10 cases and 21 controls, all dogs had call rates >90%. We removed 5,723 markers with call rates <90% from the analysis. We also removed 89,179 markers with MAF <10% and 366 markers strongly deviating from Hardy-Weinberg equilibrium in the controls (p<10^−5^). The final Irish Terrier dataset consisted of 31 dogs and 82,671 SNPs.

We performed an allelic association study and determined an empirical significance threshold by performing 100,000 permutations of each dataset with arbitrarily assigned phenotypes.

As both datasets showed considerable population stratification, we also analyzed the data using GenABEL and a mixed model approach [27]. This procedure corrects for cryptic relatedness by using the genomic kinship estimated from the marker data as covariable in the model. In both cohorts the same markers showed the highest association regardless whether the simple PLINK analysis or the mixed model GenABEL analysis was performed. With the correction for population stratification the genomic inflation factors were reduced from 1.40 to 1.07 in Kromfohrländer and from 1.32 to 1.02 in Irish Terriers. The corrected p-values (Pc1df) for the best associated markers were then 4.7×10^−8^ in Kromfohrländer and 4.2×10^−6^ in Irish Terriers.

We also used PLINK to search for extended intervals of homozygosity with shared alleles. The final critical interval was defined by visual inspection of all SNP chip genotypes on chromosome 5 for the 13 Kromfohrländer and 10 Irish Terrier cases in an Excel-file.

### Gene analysis

We used the dog CanFam 3.1 assembly for all analyses. All numbering within the canine *FAM83G* gene corresponds to the accessions XM_003434636.2 (mRNA) and XP_003434684.1 (protein). We analyzed the functional effects of variants *in silico* with SIFT, Polyphen-2 und PMUT [20]–[22].

### Whole genome sequencing of an affected Kromfohrländer

We prepared a fragment library with 300 bp insert size and collected 293,647,193 illumina HiSeq2500 paired-end reads (2×100 bp) or roughly 23.5× coverage. We mapped the reads to the dog reference genome using the Burrows-Wheeler Aligner (BWA) version 0.5.9-r16 [28] with default settings and obtained 551,317,870 uniquely mapping reads. After sorting the mapped reads by the coordinates of the sequence and merging the 2 lanes of data with Picard tools, we labeled the PCR duplicates also with Picard tools (http://sourceforge.net/projects/picard/). We used the Genome Analysis Tool Kit (GATK version v2.3-6, [29]) to perform local realignment and to produce a cleaned BAM file. Variant calls were then made with the unified genotyper module of GATK. Variant data for each sample were obtained in variant call format (version 4.0) as raw calls for all samples and sites flagged using the variant filtration module of GATK. Variant calls that failed to pass the following filters were labeled accordingly in the call set: (i) Hard to Validate MQ0 ≥4 & ((MQ0/(1.0 * DP)) >0.1); (ii) strand bias (low Quality scores) QUAL <30.0 || (Quality by depth) QD <5.0 || (homopolymer runs) HRun >5 || (strand bias) SB >0.00; (iii) SNP cluster window size 10. The snpEFF software [30] together with the CanFam 3.1 annotation was used to predict the functional effects of detected variants. We considered the following snpEFF categories of variants as non-synonymous: NON_SYNONYMOUS_CODING, CODON_DELETION, CODON_INSERTION, CODON_CHANGE_PLUS_CODON_DELETION, CODON_CHANGE_PLUS_CODON_INSERTION, FRAME_SHIFT, EXON_DELETED, START_GAINED, START_LOST, STOP_GAINED, STOP_LOST, SPLICE_SITE_ACCEPTOR, SPLICE_SITE_DONOR. The critical interval contained 610,700 bp and 15,160 coding nucleotides, respectively. In our re-sequencing data, we had ≥4× coverage on 566,516 bp of the critical interval (93%) and on all 15,160 coding bases.

Additionally, we searched for structural variations (deletions, insertions, inversions) within the critical interval using the software SVDetect [31]. SVDetect calls intrachromosomal and interchromosomal rearrangements from discordant, quality pre-filtered read pairs. As per the authors' suggestion SVDetect software was set to detect rearrangements with 3 or more supporting read pairs using 2 times standard deviation of the insert size as threshold for both deletions and duplications. This analysis identified 15 rearrangements between 100 and 450 bp in size in the critical interval. Most of these variants were within repeats and none of them affected an exon of the annotated genes in the critical interval. The sequence data of the affected Kromfohrländer were deposited in the short read archive of the European Nucleotide Archive (ENA) under accession PRJEB6076.

### Sanger sequencing

We used Sanger sequencing to confirm the illumina sequencing results and to perform targeted genotyping for selected variants. For these experiments we amplified PCR products using AmpliTaqGold360Mastermix (Applied Biosystems). PCR products were directly sequenced on an ABI 3730 capillary sequencer (Applied Biosystems) after treatment with exonuclease I and shrimp alkaline phosphatase. We analyzed the Sanger sequence data with Sequencher 5.1 (GeneCodes).

## Supporting Information

Figure S1 Expression of FAM83G in canine footpad epidermis. Immunofluorescence analysis was performed on formalin-fixed paraffin embedded sections from the paw of a non-affected control dog incubated with the FAM83G antibody NBP1-93722 (Novus Biologicals; green). Nuclei were counterstained with Hoechst 33258 (B-2883, Sigma, St-Louis, MO; blue). Conventional staining procedures were used as described [32]. The dotted line indicates the basement membrane. Note that the FAM83G antibody preferentially binds to the deep epidermis. FAM83G protein expression in the paws of this dog is consistent with *FAM83G* mRNA expression levels in the range of the abundantly expressed junctional plakoglobin (*JUP*) mRNA as detected by RNAseq (data not shown).
(TIF)

Table S1 Dogs used for whole genome sequencing.
(XLSX)

Table S2 Control dogs from other breeds.
(XLSX)
