# Supplementary figures and images for: A Mutation in the FAM83G Gene in Dogs with Hereditary Footpad Hyperkeratosis (HFH)
Source: PLoS Genet. 2014 May 15;10(5):e1004370. doi: 10.1371/journal.pgen.1004370 (PMC4022470; doi:10.1371/journal.pgen.1004370)

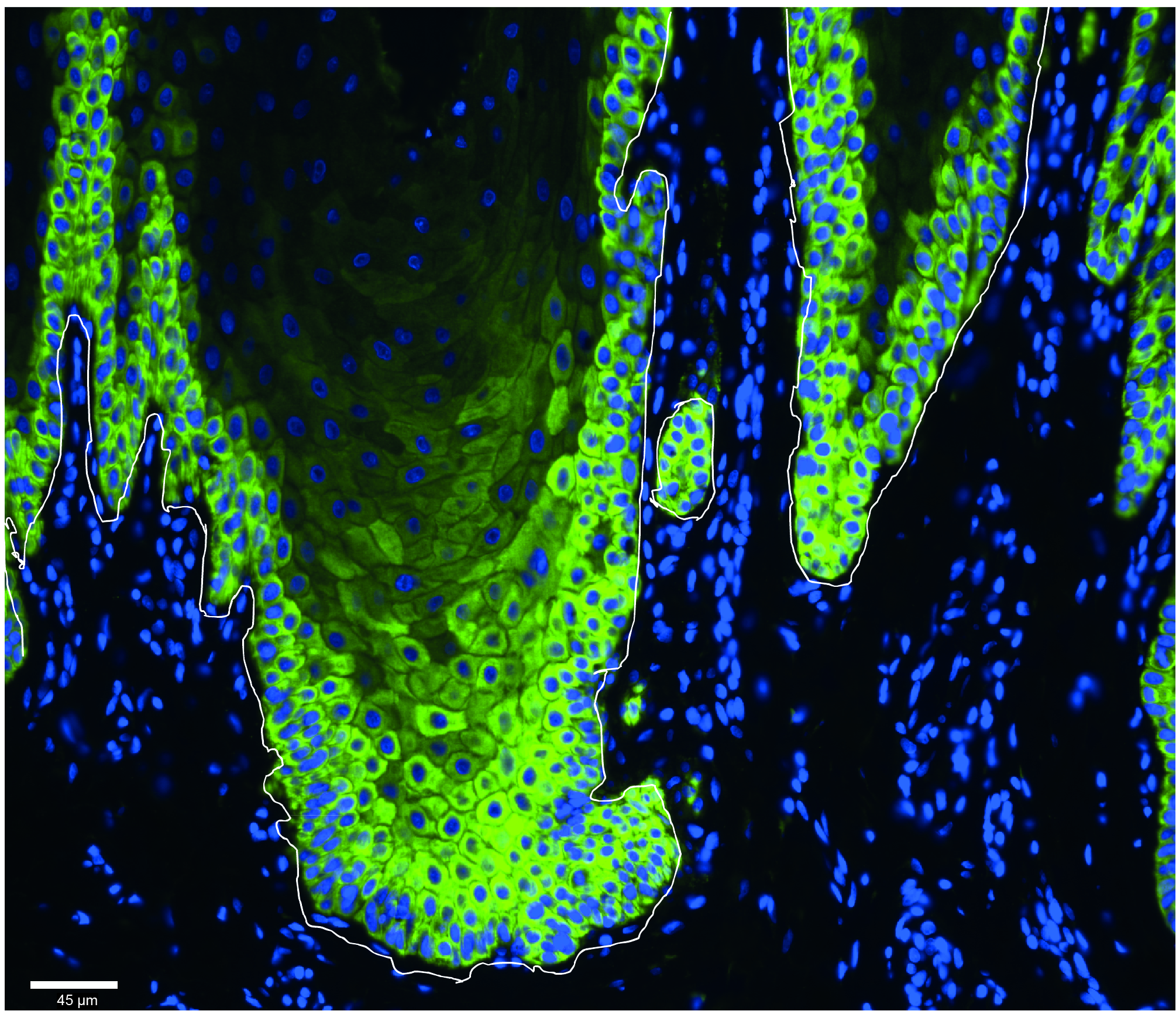

Supplement: Figure S1 — Expression of FAM83G in canine footpad epidermis. Immunofluorescence analysis was performed on formalin-fixed paraffin embedded sections from the paw of a non-affected control dog incubated with the FAM83G antibody NBP1-93722 (Novus Biologicals; green). Nuclei were counterstained with Hoechst 33258 (B-2883, Sigma, St-Louis, MO; blue). Conventional staining procedures were used as described [32]. The dotted line indicates the basement membrane. Note that the FAM83G antibody preferentially binds to the deep epidermis. FAM83G protein expression in the paws of this dog is consistent with FAM83G mRNA expression levels in the range of the abundantly expressed junctional plakoglobin (JUP) mRNA as detected by RNAseq (data not shown). (TIF) [file pgen.1004370.s001.tif]
